# Comparison of Histological Changes in Tissue Specimens Kept in Tap Water, River Water and Seawater for Different Intervals of Time

**DOI:** 10.7759/cureus.36736

**Published:** 2023-03-27

**Authors:** Mimansha Patel, Asha Saroja Peyyeti, Subhasini Singh, Manu Gupta, Mrigakshi Yadav, Renuka B, Ramanpal Singh

**Affiliations:** 1 Department of Oral Pathology and Microbiology, Triveni Institute of Dental Sciences, Hospital & Research Centre, Bilaspur, IND; 2 Department of Dentistry, Star Dental Care, Parsippany, USA; 3 Department of Oral Pathology, Awadh Dental College & Hospital, Jamshedpur, IND; 4 Department of Oral and Maxillofacial Surgery, Faculty of Dental Sciences, Shree Guru Gobind Singh Tricentenary University, Gurugram, IND; 5 Public Health and Social Work, Benestar, Melbourne, AUS; 6 Department of Orthodontics and Dentofacial Orthopaedics, Triveni Institute of Dental Sciences, Hospital & Research Centre, Bilaspur, IND; 7 Department of Oral Medicine and Radiology, New Horizon Dental College and Research Institute, Bilaspur, IND

**Keywords:** time, sea water, tap water, river water, drowning

## Abstract

Background: Forensic pathologists find it difficult to ascertain the actual reason for death and the actual mode of death in drowning cases. It is commonly acknowledged that not all people whose dead bodies are found in water perished from drowning. The medico-legal inquiry includes an important component that examines dead bodies collected from the water. The corpse's time in the water continues to be the main concern. This aids in determining the time of death, which is crucial to any medico-legal investigation. The traditional histological analysis of hematoxylin and eosin (H&E)-stained oral soft tissue can be crucial in the assessment of this feature. Hence, this study was done to compare histological changes in the tissue specimens kept in tap water, river water, and seawater for different intervals of time.

Materials and methods: One hundred eighty specimens were obtained from 180 dead bodies and divided randomly into three categories. Category A consisted of 60 specimens submerged in tap water, Category B of 60 specimens submersed in river water, and Category C consisted of 60 specimens submerged in seawater. The specimens underwent routine histological processing and H&E staining. The microscopic evaluation of specimens was carried out at two hours and on the first, second, third, fourth, and fifth days of submersion. Details were recorded regarding the structural changes, cellular changes, connective tissue changes, changes in the basement membrane, and intensity of H&E staining. Once the process of routine histological processing and H&E staining was completed for each study specimen, an evaluation of microscopic changes in the tissue was made.

Results: The present study revealed that seawater preserved tissue best and for a longer period than river water and tap water. River water preserved tissue better than tap water. In pathologic tissue, details were preserved for much longer. It was noted that in squamous cell carcinoma, connective tissue was destroyed earlier than epithelium, in contrast to normal tissue where epithelium was destroyed before connective tissue.

Conclusion: This study indicates that the medium of submersion in drowning victims affects the histopathological features. The time of death is an important aspect of forensic investigation. Considering this, all cases of drowning should be very carefully evaluated, and the medium taken into consideration while determining the time of death, as tissue degrades faster in freshwater than in seawater.

## Introduction

Forensic pathologists find it difficult to ascertain the actual cause and mode of death in drowning instances. It is commonly acknowledged that not all people whose dead bodies are found in water perished from drowning. Dead bodies hauled up from water may have suffered death from natural diseases, catastrophes that occurred before or while they were submerged in the water, or consequences of submersion other than drowning [1,2]. Lunetta et al. explained that while examining the incidents mentioned above may reveal traces of submersion, this merely serves to establish that they might have been inside water [3]. It does not help distinguish between different death modes or establish the correct cause of death [3,4]. The inhalation of the drowning media into the airways is one of the principal drowning-related positive results. These results, however, are not conclusive because these findings can also be noticed in other circumstances (drug intoxication, cardiovascular disorder, etc.). Consequently, a full autopsy should always be conducted in cases of total immersion in water to classify (and consequently explain adequately) all injury issues present, to ascertain whether or not the victim died after being drowned, as well as to check for any possible natural ailments, such as cardiovascular events, cerebrovascular disorders, and hypertensive disorders that are pathological in the form of heart disorders due atheroclerosis, infarction, that may have contributed to, prompted, or even caused the victim's death [5,6]. To ascertain whether the dead seemed to be under the impact of alcohol and other substances, a thorough toxicological assessment at the moment of death is also crucial. The co-evaluation of credible evidence is crucial in uncertain circumstances while determining the reason (sinking, natural illness, etc.) and the mode of death (accident, suicide, or homicide) [7,8].

Lunetta et al. offer an intriguing analysis of potential macroscopical results in a significant number of drowning cases [3]. During an external inspection, the frothy plume on the lips and nose might be regarded as an important indicator. The drawback is that this discovery is non-specific, fleeting, and limited to recently drowned corpses. Additionally, none of the other symptoms are diagnostic for drowning but are immersion symptoms, rather [9-12]. The pathologist must consider drowning-related death mechanisms to correctly evaluate the autopsy results. Submersion is when the head is also submerged in water. The specific processes after drowning in humans are still unknown, despite extensive research into the chronology of activities following submersion in animal studies. For long, it was assumed that 85%-95% of people who have died from water aspiration exhibit drowning-related symptoms, while the remaining most likely pass away from vagal suppression, post-immersion sickness, or laryngeal contraction [13,14]. The occurrence and significance of laryngospasm are still debatable. In fact, drowning almost certainly never happens even without any degree of aspiration. In addition, hypothermia can develop quickly in people who are swimming or struggling to stay on the surface of cold water, particularly after a catastrophe in open seawater [15,16]. Medico-legal inquiry includes an important component that examines dead bodies collected from the water. The dead body's time in the water continues to be a major concern. This aids in determining the time of death, which is crucial to any medico-legal investigation. Traditional histological analysis of hematoxylin and eosin (H&E)-stained oral soft tissue can be crucial in the assessment of this feature [17,18]. To the best of the authors' knowledge, no study has ever been done examining the histological changes in oral soft tissue as proof in cases of death due to drowning. This research, therefore, was conducted to compare histological changes in the oral tissue specimens kept in tap water, river water, and seawater for different intervals of time.

## Materials and methods

**Figure 1:**
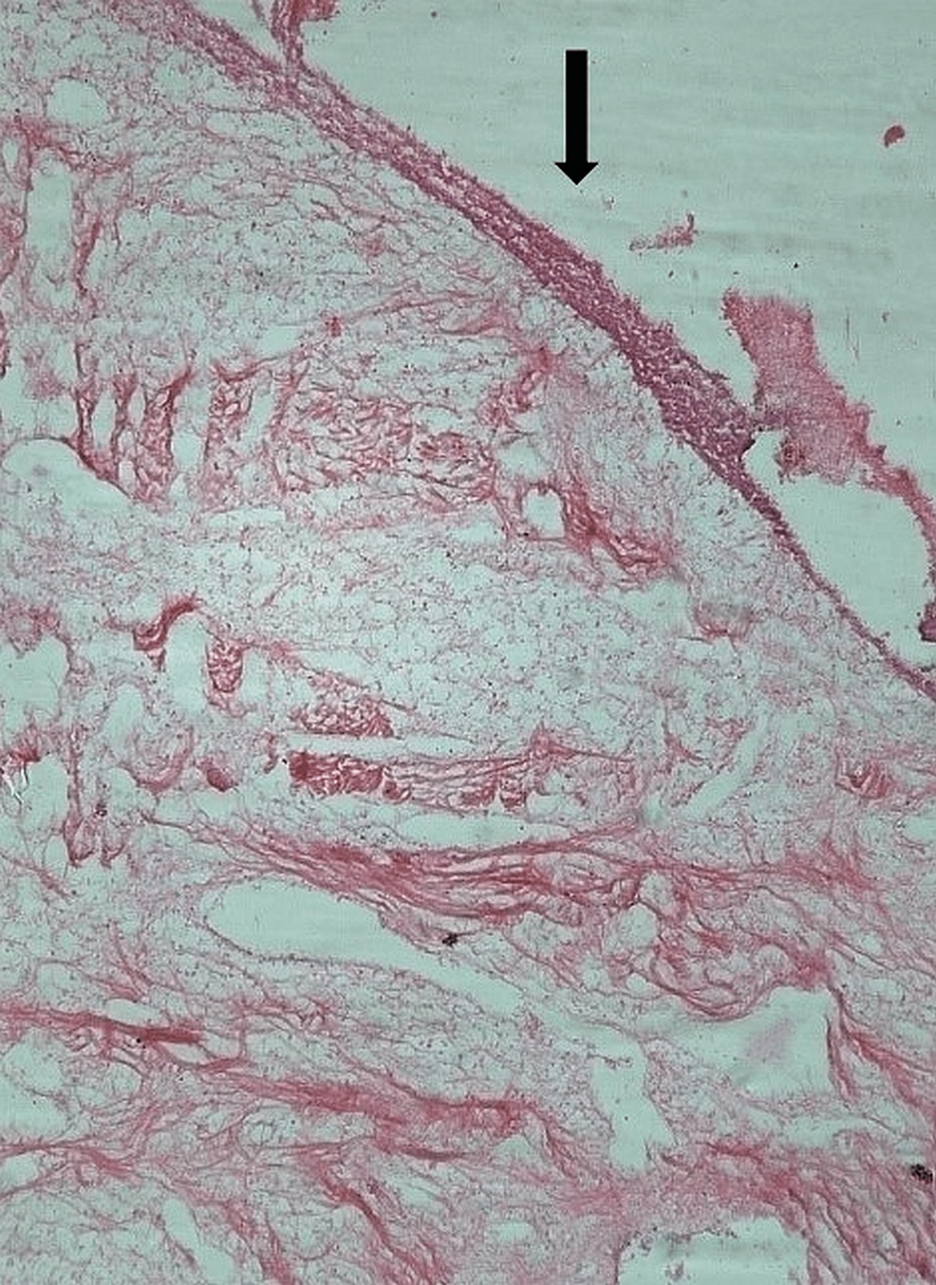
Histological representation of normal tissue (arrow)

One hundred eighty oral soft tissue specimens were obtained from 60 dead bodies, with ethical clearance from the Triveni Dental College, Bilaspur, Ethical Committee (IRB number IEC/TRIVENI/2020/111). Dead bodies' submersion time remains the biggest concern. The exact time of death is crucial for medico-legal examinations. To examine this, the 180 specimens were divided randomly into three categories. Category A consisted of 60 specimens submerged in tap water (37°C), Category B of 60 specimens submersed in river water (37°C), and Category C of 60 specimens submerged in seawater (37°C). The specimens underwent routine histological processing and H&E staining. The microscopic evaluation of the specimens was carried out at two hours and, the first, second, third, fourth, and fifth days of submersion. Details were recorded according to the Sign Grading System, regarding the structural, cellular, and connective tissue changes, changes in the basement membrane, and intensity of H&E staining, where normal tissue representation is shown in Figure 1 [18].

**Figure 2:**
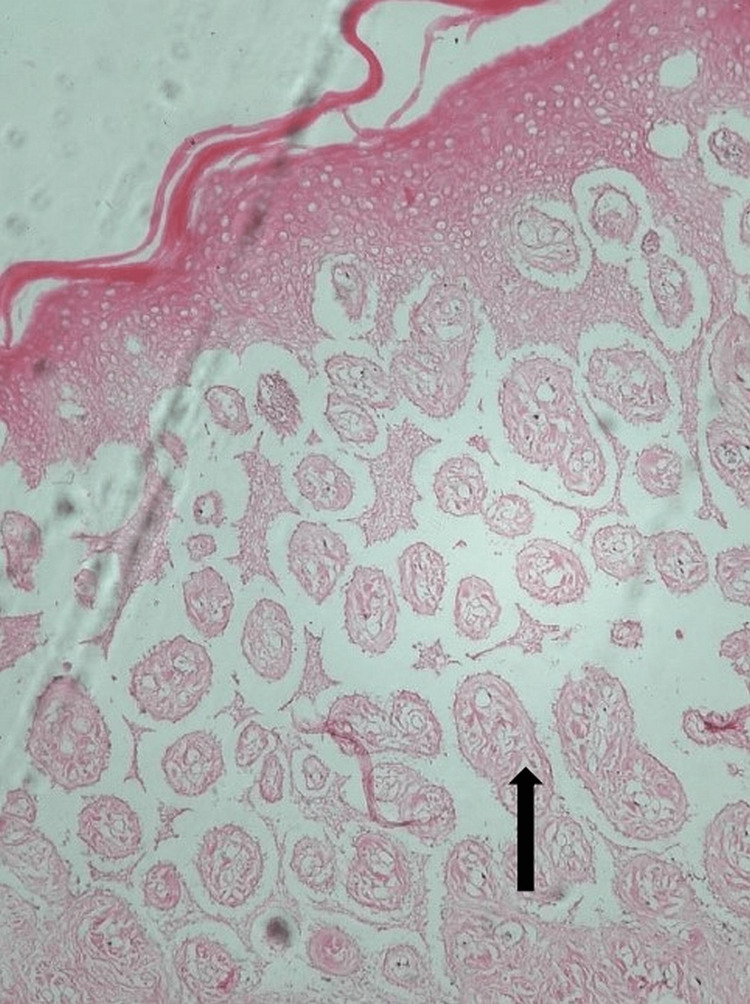
Representation of tissue destruction when immersed for five days in river water (arrow)

A representation of destructive tissue when immersed for five days in river water is shown in Figure 2.

Routine histological processing

The fresh tissue samples were extracted from dead bodies. As quickly as practicable after dissection, bodies were handled professionally and fixed, as needed. Fixation ideally happened at the location of removal, either in the operating room or right after their transport to the lab. A fixing agent in a liquid state (fixative), like formaldehyde solution (popularly known as formalin), was used to fix the specimen. Preferably, specimens were immersed in a fixative for sufficient time to allow the fixative to reach each area of the tissue specimen and for an extended amount of time to permit the fixation's chemical reactions to achieve equilibrium. The specimen was fixed for 6-24 hours. The specimens may need additional dissection after fixation for choosing the best places to examine. The treated specimens were put in appropriately labeled cassettes (little perforated baskets) to distinguish them from each other. The major portion of the water inside a tissue specimen must be eliminated to enable proper infusion of molten wax because molten wax is not soluble in water due to its hydrophobic nature. Commonly, samples were submerged in a sequence of different ethanol solutions with progressively higher concentrations until they were exposed to pure alcohol without water. Since ethanol and water mix in all quantities, the water in the tissue specimen gradually gives way to alcohol. To prevent severe tissue distortion, a succession of escalating concentrations was used. Now, the tissue has to be penetrated with an appropriate histological wax. Histology waxes based on paraffin wax are used most often, despite many other chemicals having been tested and employed for this function over the ages. When cooled down to 20°C, normal wax hardens to a firmness that makes it possible to cut portions of tissue uniformly. A standard wax is molten at a temperature of 60°C and could be injected into tissues at this temperature. The physical traits of these wax preparations enable tissues infused with the wax preparations to be sectioned properly at a layer with a thickness of at least 2 μm. The tissue specimen was shaped into a "block" that could be inserted into a microtome for segment cutting, after being fully infiltrated with wax. An "embedding center" was used to complete this stage, where molten wax was poured into a mold before the specimen was put inside. The orientation of the specimen in the mold was done with great care since it would affect the "plane of the section". The mold was covered with a cassette, filled with additional wax, and set on a cooling surface to harden.

Steps of H&E staining

All the components were covered in and infiltrated with paraffin wax, which is usually hydrophobic and hence resistant to water-based chemicals, after the adequate production of a paraffin section. Most of the components of cells and tissues lack natural color and cannot be seen. Dissolving all the wax with xylene was the first process in conducting an H&E staining. After full de-waxing, the slide was properly rinsed in water before being put through substantial concentrations of alcohol to eliminate the chemical xylene. Now that the section was moist, aqueous chemicals could easily permeate the components of the tissue. The prepared slide was now stained with the help of a nuclear stain, namely, Harris hematoxylin, which contains an aluminum salt as a binding agent and oxidized hematoxylin or hematein as a dye. Consequently, the nuclei and several other components first took on a reddish-purple hue. The tissue section was "blued" by processing with a slightly alkaline solution after being rinsed in tap water. Hematoxylin turned dark blue at this stage. After cleaning the section, it may be examined to determine how well-stained the nuclei are, whether they contrast enough, and how much background stain is present. When Harris hematoxylin was used, a de-staining step was typically necessary to get rid of background staining that was not specific and to boost contrast. A mild acid alcohol is used. Following this procedure, thorough rinsing and blueing are once more necessary. Regressive stains are staining procedures involving a de-staining or differentiating stage. A solution of eosin in water or alcohol was now used to stain the segment. This gives a variety of non-nuclear elements a pink hue. After the application of the eosin stain, the prepared slide was repeatedly washed in xylene baths to "clear" the tissue and make it entirely transparent. This process removes all remaining water from the slide. A thinner layer of polystyrene mount was placed, and a coverslip of glass was placed on top. Once the process of routine histological processing and H&E stain was completed for each study specimen, microscopic changes in the tissue were evaluated.

SPSS software, version 21 (IBM Corp., Armonk, NY) was used for statistical analysis. A comparison of staining criteria and microscopic details was carried out using the Kruskal-Wallis test with a p-value < 0.05 as significant.

## Results

When the specimens drowned in tap water were analyzed, the degree of separation of epithelium and connective tissue was (+/-, partially present) at two hours after submersion. The degree of separation of epithelium and connective tissue was (++, increased) on the first, second, third, fourth, and fifth day after submersion. The degree of acanthosis was (+, slightly present) at two hours of drowning and (++) on the first, second, third, fourth, and fifth day after submersion. The degree of ballooning of cells was (+) at two hours and the second day of drowning, while it was (++) on the first, second, third, fourth, and fifth day of drowning. The degree of nuclear fragmentation was (-, not seen/absent) in the second hour while it was (++) on the fourth and fifth day. The nuclear fragmentation was not distinguishable on the first, second, and third day. The degree of changes in connective tissue fibers at the second hour was (+/-) and it was (+) on the second day. There were findings of separation in connective tissue fibers on the first, second, third, fourth, and fifth day. The degree of changes in muscle fibers was (+, slight changes) at two hours and on the first day. No changes were observed on the second day, while separation was observed on the third, fourth, and fifth day. Separation was observed in the basement membrane at the second hour, and on the third, fourth, and fifth day while it was indistinguishable on the first day, and fragmentation was observed on the second day. The intensity of the H stain was patchy at the second hour, faint on the first day, and (-) on the second, third, fourth, and fifth day. The intensity of the E stain was (++, more) at the second hour, while it was (+) on the first, second, and third day, and (-) on the fourth and fifth day (Table 1).

**Table 1:** Changes observed in specimens kept in tap water CT: connective tissue; H: hematoxylin; E: eosin +/- signifies some sample changes seen and some not seen; ++ signifies increased stain; + signifies light stain; - signifies changes not seen

S.no.	Sample	Structural changes		Cellular changes		CT changes		Basement membrane	Intensity	
		Separation – epithelium and CT	Acanthosis	Ballooning of cells	Nuclear fragmentation	CT fibres	Muscles		H	E
1.	2 hours	+/-	+	+	-	-/+	+	Separation	Patchy	++
2.	1st day	++	++	++	Not visible	Separation	+	Indistinguishable	Faint	+
3.	2nd day	++	++	+	Not visible	+	No change	Fragmented	-	+
4.	3rd day	++	++	++	Indistinguishable	Separation	Separation	Separation	-	+
5.	4th day	++	++	++	++	Separation	Separation	Separation	-	-
6.	5th day	++	++	++	++	Separation	Separation	Separation	-	-

Microscopic evaluation of specimens in river water revealed no changes in nuclear fragmentation at the second hour of drowning, while it was (+) on the first, second and third day and (++) on the fourth and fifth day. Loosely arranged collagen was observed on the second and third day, while separation of connective tissue fibers was observed at the second hour, and on the first, fourth and fifth day. No changes were observed in muscles at the second hour, and on the first, second, and third day, while separation was observed on the fourth and fifth day. Basement membrane separation was observed at the second hour, and on the second, third, fourth and fifth day. The intensity of H stain was (-) at most of the time intervals, while that of E stain was patchy on the third day (Table 2).

**Table 2:** Changes observed in specimens kept in river water CT: connective tissue; H: hematoxylin; E: eosin +/- signifies some sample changes seen and some not seen; ++ signifies increased stain; + signifies light stain; - signifies changes not seen

S.no.	Sample	Structural changes		Cellular changes		CT changes		Basement membrane	Intensity	
		Separation – epithelium and CT	Acanthosis	Ballooning of cells	Nuclear fragmentation	CT fibers	Muscles		H	E
1.	2 hours	+	-	+	No changes	Separation	No change	Separation	+	++
2.	1st day	+	+	+	+	Separation	No change	+/-	+	+
3.	2nd day	++	+	++	+	Loosely arranged collagen	No change	Separation	-	+
4.	3rd day	++	++	++	+	Loosely arranged collagen	No change	Separation	-	Patchy
5.	4th day	++	++	++	++	Separation	Separation	Separation	--	-
6.	5th day	++	++	++	++	Separation	Separation	Separation	--	-

**Figure 3:**
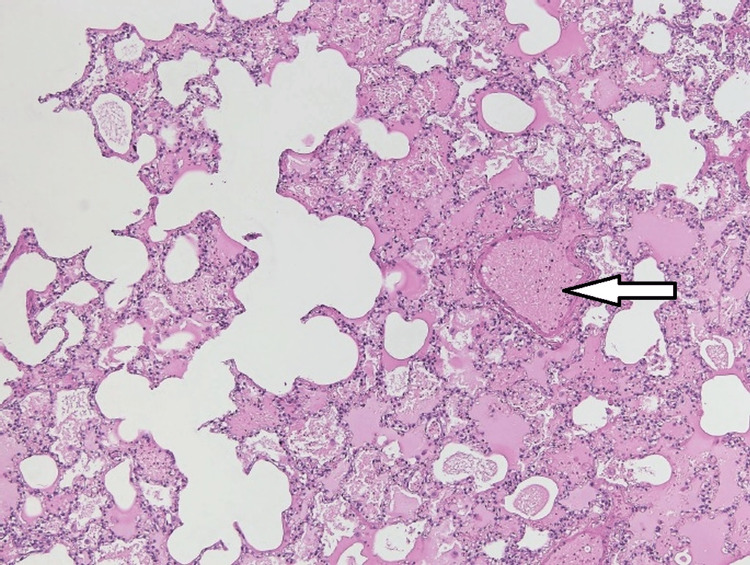
Histological image of the changes in epithelium after five days The arrow shows degenerative changes.

Analysis of specimens drowned in seawater revealed the epithelium and connective tissue to be well preserved on the second day of drowning, but there were no changes in nuclear fragmentation. In the case of connective tissue fibers, separation was commonly observed on the first, second, third, fourth, and fifth day. In muscles, the changes were faint on the first, second, third, fourth and fifth day. There were no changes in the basement membrane at the second hour, and on the first, second and third day. The intensity of H stain was (+++, strong) at the second hour, (++) on the first day and (+) on the second, third, fourth and fifth day. The intensity of E satin was (++) at the second hour and (+) on the first, second, third, fourth and fifth day (Table 3; Figure 3).

**Table 3:** Changes observed in specimens kept in seawater CT: connective tissue; H: hematoxylin; E: eosin +/- signifies some sample changes seen and some not seen; +++ signifies strong stain; ++ signifies increased stain; + signifies light stain; - signifies changes not seen

S.no.	Sample	Structural changes		Cellular changes		CT changes		Basement membrane	Intensity	
		Separation – epithelium and CT	Acanthosis	Ballooning of cells	Nuclear fragmentation	CT fibers	Muscles		H	E
1.	2 hours	Intraepithelial	-	-	+/-	No change	No change	No changes	+++	++
2.	1st day	++	+	++	+	Separation	Faint	No changes	++	+
3.	2nd day	Well-preserved	+	-	No change	Separation	Faint	No changes	+	+
4.	3rd day	++	+	+	+	Separation	Faint	No changes	+	+
5.	4th day	++	++	++	++	Separation	Faint	Separation	+	+
6.	5th day	++	++	++	++	Separation	Faint	Separation	+	+

The present study revealed that seawater preserved the tissue best and longer than river water and tap water. River water preserved the tissue better than tap water. In pathological tissue, details, e.g. structural changes, were preserved for a much longer period. It was noted that in structural changes, connective tissue was destroyed earlier than epithelium, in contrast to normal tissue where epithelium was destroyed before connective tissue. It was observed that histological changes that occurred in the tissue depend on two factors: the type of medium of submersion and the time elapsed since death (p ≤ 0.005) (Table 4).

**Table 4:** Descriptive data analysis

Variables	Mean ranks	p value
Time elapsed since death	22.22	0.0002
Type of medium of submersion	15.21	0.0001

## Discussion

Investigations into bodies found in water comprise a significant portion of medico-legal investigations. According to published research, one of the most challenging forensic medicine diagnoses is drowning. Acute drowning is characterized by hypoxemia and irreversible cerebral anoxia and is defined as death brought on by immersion in a liquid. There have been endeavors to determine the precise moment of death because this is crucial to forensic inquiry [19]. There have been instances where the location of the death was different from the location where the body was found. The victim is frequently killed on land and the body is dumped in the water. Therefore, it will be simpler to determine the precise time of death if there is unambiguous documentation of the change in the soft tissue linked to the amount of time elapsed after immersion in various media [20]. Despite substantial research into the timeline of events following immersion in animal studies, the precise mechanisms after drowning in humans remain unknown. For a very long time, it was believed that 85%-95% of those who die from water aspiration show symptoms of drowning, and the remaining 3% or more most likely die from vagal suppression, post-immersion illness, or laryngeal contraction. Both the occurrence and the significance of laryngospasm are still subject matters of debate. Drowning is extremely unlikely to occur even in the absence of any aspiration [21,22].

Additionally, hypothermia can strike fast in persons swimming or straining to stay above cold water, especially following an accident in open seawater. An essential part of the medico-legal investigation consists of looking at deceased bodies pulled out of water. The primary worry is still how long the corpse was submerged. In any medico-legal examination, knowing the exact moment of death is essential [23,24]. The evaluation of this feature may require a traditional histological examination of the oral soft tissue stained with H&E. To the best of the authors' knowledge, there has never been a study that looked at the histological changes in the oral soft tissue as evidence in cases of drowning death [25,26]. The present study revealed that seawater preserved tissue best and longer than river water and tap water. This is because of (a) a high fluoride content (1.3 mg/l), (b) bacteriostatic and bactericidal activity and (c) a high NaCl content (hypertonic 3%). River water preserves tissue better than tap water, as it is running and not stagnant. It, therefore, becomes pertinent to know the sequence of tissue changes as seen by microscopy. Subtle differences in the changes associated with time could point to the source of drowning. Thus, it is very important to pinpoint the scene of the death. One of the main drowning-specific findings is the inhalation of the drowning media into the respiratory airways. However, these observations are not considered definitive because they can also be noticed in some other circumstances (severe drug abuse, cardiovascular disorder), and the absence of inhalation of the drowning media inside respiratory airways does not completely rule out drowning [27-30]. A thorough toxicological evaluation at the time of death is also essential to determine whether the deceased was under the influence of alcohol or other drugs. When deciding the cause of death and the method of death, it is critical to evaluate trustworthy evidence concurrently [31,32].

In our investigation, it was found that features were retained for a considerably longer time in the pathological tissue, such as squamous cell carcinoma (SCC). It is tempting to conclude that the tissue is possibly somewhat impermeable to the medium because of the alterations brought on by cancer in the tissue. In contrast to the normal tissue, where the epithelium is destroyed before the connective tissue, it was found that in SCC, the connective tissue is damaged first. Even after extensive tissue loss, the melanin pigment is unharmed. The ramifications of these findings cannot be explained by melanin's recognized properties. It was observed that histological changes that occur in the tissue depend on two factors: the type of medium of submersion and time elapsed since death. A fascinating examination of possible macroscopical outcomes in a considerable proportion of drowning cases has been provided by researchers. During an external inspection, the foamy plume on the lips and nose can be considered a crucial sign. The disadvantage is that this finding is general, transient, and restricted to newly drowned bodies. Furthermore, none of the additional symptoms are indicative of drowning but are signs of immersion. To properly assess the findings of the autopsy, the pathologist must consider drowning-related death mechanisms [33,34].

This study was not without limitations. Only histological changes were assessed, though clinical representations should also be diagnosed. The sample size taken was small. Also, different temperatures could be a limitation because 37 degrees is too high for seawater, but the same is essential for a good study.

## Conclusions

Researchers in the forensic dentistry field have been looking for diagnostic approaches that give equal weight to all of the body tissues and can be used for a wide range of medico-legal objectives. Tissue in the presence of any fluid degrades fast and hence has to be carefully studied. This study indicates that the medium of submersion in drowning victims affects the histopathological features. The time of death is an important aspect of a forensic investigation; while considering this, it is important to note that tissue degrades faster in freshwater than in seawater, and hence, all cases of drowning should be evaluated very carefully. The medium should be taken into account while determining the time of death.
